# A Case of Fulminant Infective Endocarditis in Mitral Valve Prolapse With Septic Emboli and an Annular Abscess

**DOI:** 10.7759/cureus.108806

**Published:** 2026-05-13

**Authors:** Riley L Smith, Polsha Jules, Ryan Clark

**Affiliations:** 1 Department of Internal Medicine, Liberty University College of Osteopathic Medicine (LUCOM), Lynchburg, USA; 2 Department of Cardiology, Kettering Health Network, Dayton, USA

**Keywords:** annular abscess, antibiotic policies and guidelines, cardiac abscess, dental extraction, endocarditis, infective endocarditis, mitral valve prolapse, mitral valve replacement, septic emboli

## Abstract

Mitral valve prolapse (MVP) is associated with an increased risk of infective endocarditis (IE), particularly in patients with mitral regurgitation and leaflet thickening. Historically, a subset of patients with mitral valve prolapse were considered for antibiotic prophylaxis before dental procedures. Conversely, current recommendations limit prophylaxis to the highest-risk cardiac conditions, such as congenital heart defects, mechanical valves, heart transplant with valvular issues, and a history of IE.

We present a case of a 59-year-old man with a history of MVP with mildly thickened posterior leaflet, hypertension, hyperlipidemia, and prior tobacco use who presented to the hospital with nausea and vomiting four weeks after a tooth extraction. Initial laboratory evaluation was notable for elevated inflammatory markers, elevated transaminases, hyperbilirubinemia, and elevated troponin levels. Blood cultures grew methicillin-sensitive *Staphylococcus aureus *(MSSA). Transthoracic echocardiography demonstrated MVP with new moderate mitral regurgitation. Brain MRI showed multifocal embolic infarcts and a small subarachnoid hemorrhage. Transesophageal echocardiography (TEE) revealed vegetations involving both mitral valve leaflets, extension into the mitral annulus and left atrial wall, and annular abscess formation.

The patient's hospital course was complicated by septic shock, respiratory failure, intermittent ventricular tachycardia, transient complete heart block, acute tubular necrosis, postoperative atrial fibrillation, and new-onset cardiomyopathy. He ultimately required urgent mitral valve replacement with extensive debridement of the mitral annulus and left atrial wall, followed by prolonged intravenous antibiotic therapy. Follow-up imaging showed a well-seated prosthetic valve without recurrent vegetation.

This case highlights the severe complications of IE in patients with MVP and mildly thickened leaflets following dental procedures. Current guidelines do not recommend routine prophylaxis in this population. This case raises important questions regarding whether selected moderate-risk MVP patients with leaflet thickening and mitral regurgitation may benefit from individualized prophylactic strategies.

## Introduction

Mitral valve prolapse (MVP) affects approximately 1% to 2% of the population based on contemporary echocardiographic criteria. [1,2]. MVP is defined by systolic displacement of one or both mitral valve leaflets at least 2 mm above the mitral annular plane on parasternal long-axis imaging. Although many patients have a benign clinical course, MVP is associated with an increased risk of complications, including mitral regurgitation, arrhythmias, heart failure, and infective endocarditis (IE) [1].

Patients with MVP have an estimated seven-fold greater risk of IE compared to the general population [3]. Prior studies have demonstrated that patients with leaflet thickening of 5 mm or greater and associated mitral regurgitation are at particularly elevated risk for valvular complications and IE, both of which the patient had. The 2006 American College of Cardiology/American Heart Association (ACC/AHA) valvular guidelines recommended antibiotic prophylaxis before dental procedures in patients with MVP with mitral regurgitation or thickened leaflets [4]. However, the 2007 AHA IE prevention guidelines significantly restricted prophylaxis recommendations to only the highest-risk cardiac conditions, excluding MVP [5]. This approach has been reaffirmed in more recent AHA guidance in 2021 [6,7].

Although current recommendations emphasize good oral hygiene and routine dental care for moderate-risk patients, concern remains regarding whether selected MVP patients with leaflet thickening and mitral regurgitation may still be at a clinically significant increased risk for IE after invasive dental procedures. We present a case of severe methicillin-sensitive *Staphylococcus aureus* (MSSA) IE in a patient with MVP and mildly thickened mitral valve leaflets following recent tooth extraction. MSSA is commonly isolated from the mouth, tongue, and throat.

## Case presentation

A 59-year-old male with a past medical history of MVP with mildly thickened posterior leaflet (5 mm), hypertension, hyperlipidemia, and former tobacco use presented to the emergency department with nausea and vomiting for four days. His spouse reported that he had undergone a tooth extraction approximately four weeks before presentation.

On arrival, the patient was hemodynamically stable with a late systolic murmur and a mid-systolic click. Initial laboratory evaluation demonstrated elevated troponin levels of 512 ng/L and 965 ng/L, prompting transfer to a tertiary care center for concern of non-ST elevation myocardial infarction. Additional laboratory findings were notable for the findings in Table 1.

**Table 1:** Initial laboratory findings on presentation.

Laboratory Test	Patient Value	Reference Range
Troponin I	512 ng/L, then 965 ng/L	<34 ng/L
C-reactive protein	280 mg/L	<10 mg/L
Erythrocyte sedimentation rate	53 mm/hr	0-20 mm/hr
Procalcitonin	4.89 ng/mL	<0.10 ng/mL
Aspartate aminotransferase	270 U/L	10-40 U/L
Alanine aminotransferase	145 U/L	7-56 U/L
Total bilirubin	5.6 mg/dL	0.1-1.2 mg/dL
Direct bilirubin	3.05 mg/dL	0.0-0.3 mg/dL
White blood cell count	10.7 × 10^9/L	4.0-11.0 × 10^9/L

The patient was started on empiric antibiotics of vancomycin and piperacillin-tazobactam. Blood cultures subsequently grew MSSA. Despite clinically appropriate antibiotic therapy, repeat blood cultures remained positive after two days, suggesting persistent bacteremia.

Transthoracic echocardiography demonstrated the patient’s known MVP with new moderate mitral regurgitation and preserved left ventricular ejection fraction. Due to concern for IE, transesophageal echocardiography (TEE) was obtained.

**Figure 1:**
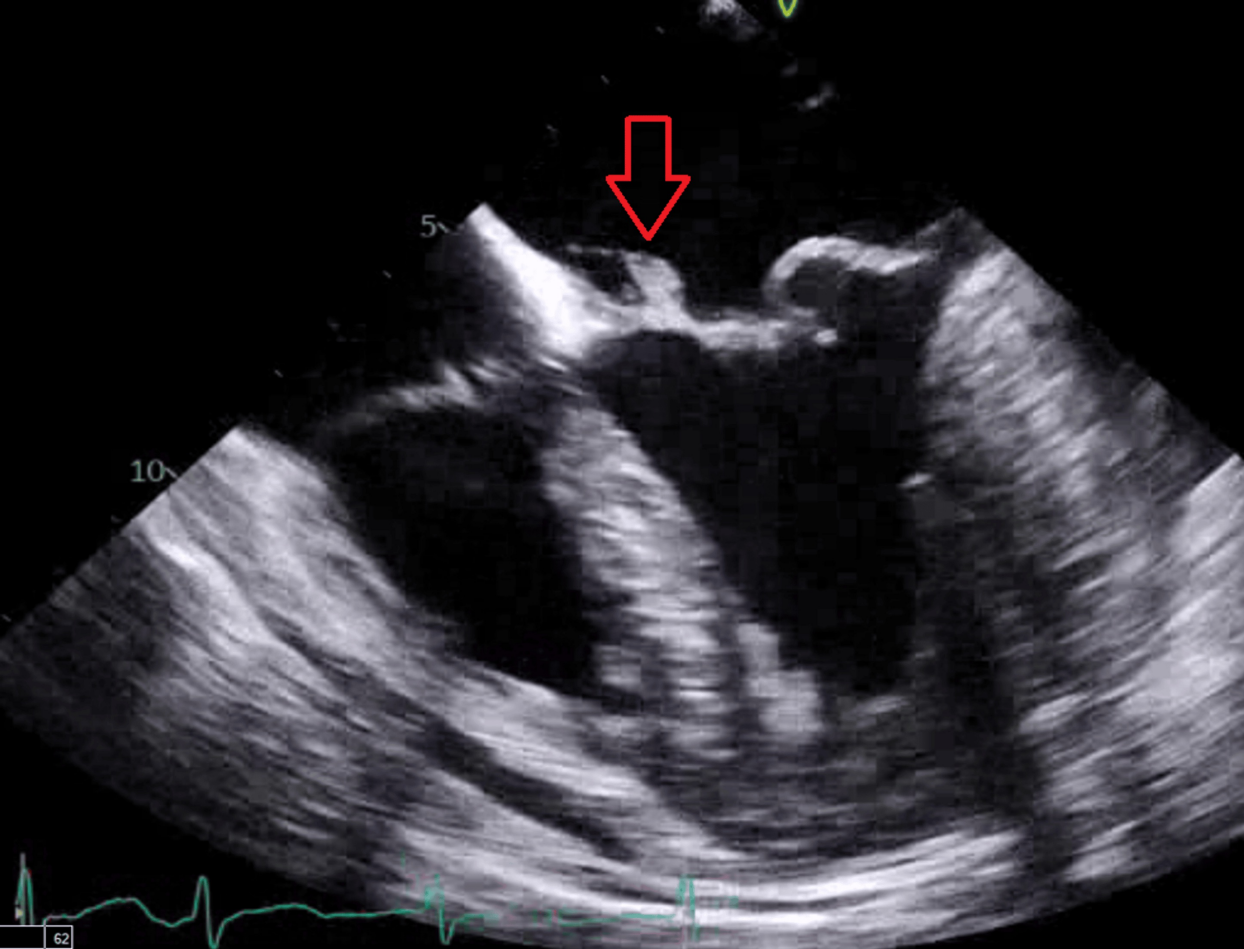
New vegetation on the mitral valve seen on transesophageal echocardiography. The red arrow indicates a new digitation on the mitral valve.

**Figure 2:**
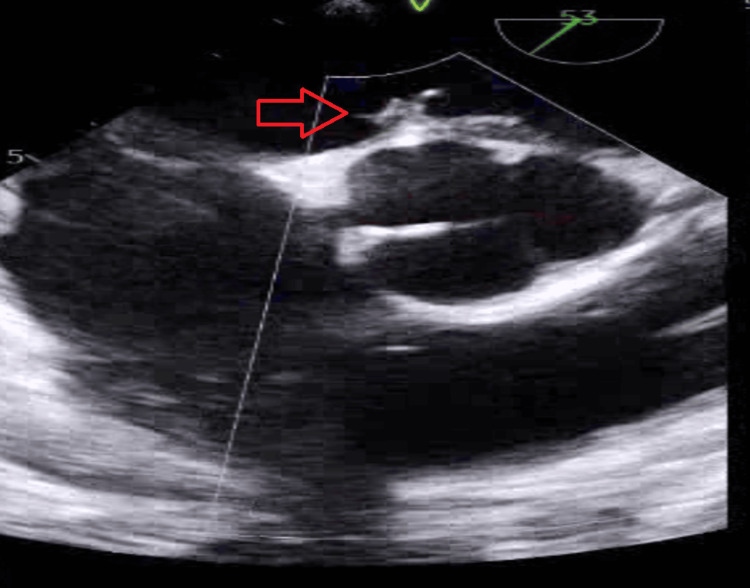
Initial transesophageal echocardiography (TEE) image from the hospitalization. The arrow demonstrates a second infective vegetation on the left atrial wall extending into the aortic valve annulus on TEE.

**Figure 3:**
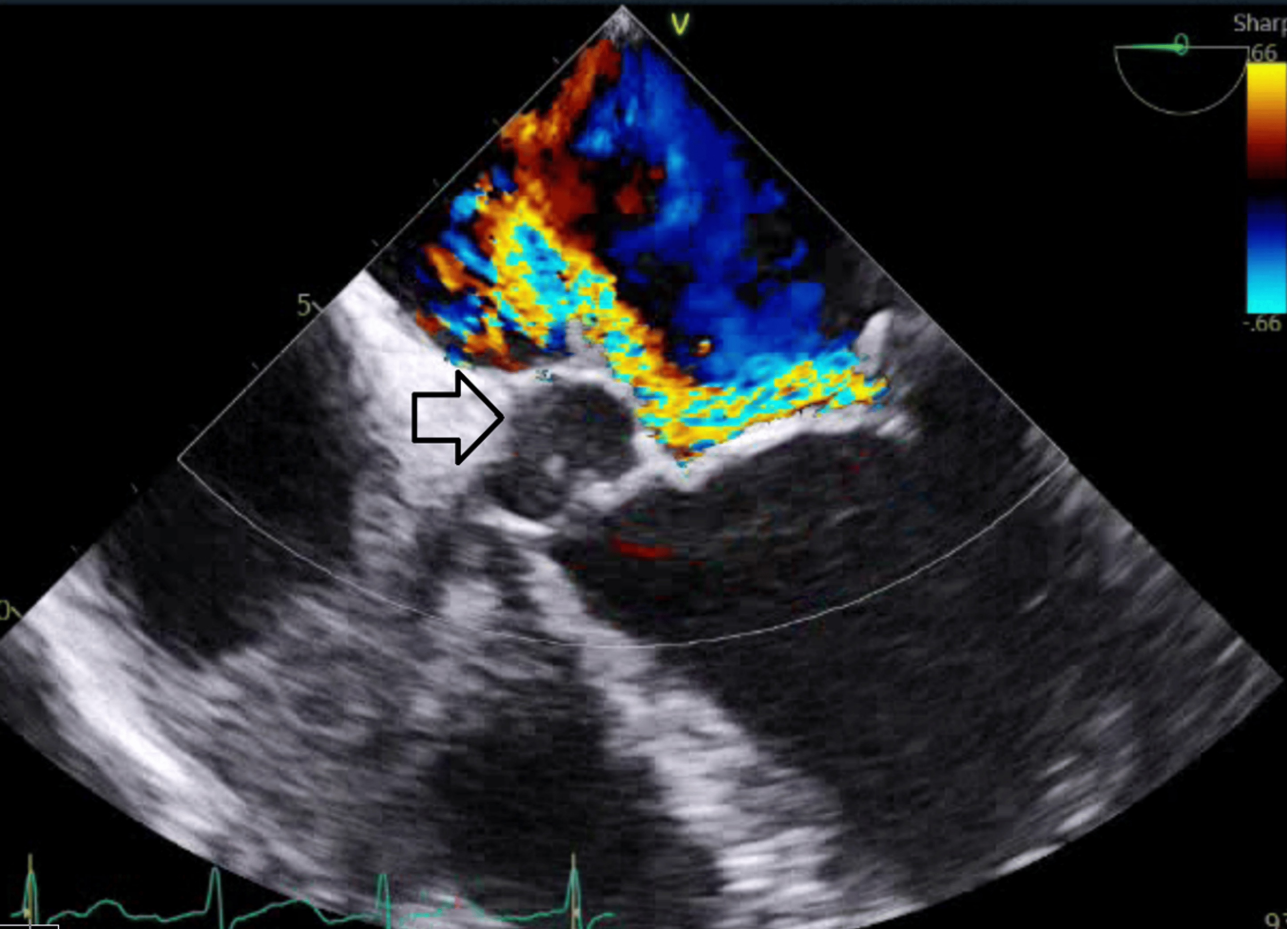
Initial transesophageal echocardiography (TEE) image from hospitalization. The arrow demonstrates the abscess in the mitral valve annulus on TEE.

TEE revealed vegetations on the atrial side of both the anterior and posterior mitral valve leaflets, with extension into the mitral annulus near the base of the interatrial septum and additional vegetation involving the left atrial wall, as seen in Figures 1, 2. Abscess formation was also present within the mitral valve annulus, which can be seen in Figure 3.

In the setting of visual hallucinations and acute encephalopathy, magnetic resonance imaging of the brain was also performed and demonstrated multifocal acute infarcts in multiple vascular territories with a small subarachnoid hemorrhage, concerning for septic emboli. Imaging additionally demonstrated periodontal disease without abscess, suggesting a possible odontogenic source. While in the intensive care unit, the patient developed respiratory failure requiring intubation. Multiple intermittent conduction abnormalities, such as intermittent nonsustained ventricular tachycardia, bradycardia, and transient episodes of complete heart block due to extension of infection into the aortic root. Cardiothoracic surgery was consulted emergently, and the patient underwent urgent mitral valve replacement, placement of a 45 mm atrial appendage clip, aortic root exploration, and extensive debridement of the left atrial wall and mitral annular abscess.

The postoperative course was complicated by severe acute tubular necrosis secondary to septic shock and vasopressor use, postoperative atrial fibrillation initially requiring intravenous amiodarone, and new cardiomyopathy with a reduced ejection fraction of 35%. He was ultimately transitioned to oral amiodarone and guideline-directed medical therapy. A peripherally inserted central catheter was placed for an extended course of intravenous cefazolin for outpatient use.

The patient was discharged to an extended rehabilitation facility to complete intravenous antibiotic therapy. At follow-up with cardiology, repeat TEE demonstrated no evidence of recurrent vegetation or endocarditis, a well-seated prosthetic mitral valve, mildly reduced left ventricular ejection fraction, and a well-seated atrial appendage clip. Left heart catheterization performed for evaluation of systolic dysfunction demonstrated 50% stenosis of the mid-left anterior descending artery, which limited flow further, in addition to the septic shock vasodilation leading to myocardial ischemia.

## Discussion

This case illustrates the potentially devastating consequences of IE in patients with MVP despite current prophylaxis recommendations. Our patient had known MVP with mildly thickened leaflets and developed severe MSSA endocarditis after a recent tooth extraction, ultimately requiring urgent mitral valve replacement and extensive debridement of periannular infection.

Historically, antibiotic prophylaxis was recommended for patients with MVP and mitral regurgitation or leaflet thickening undergoing invasive dental procedures [4]. The guidelines are based on evidence demonstrating that patients with classic MVP, defined by leaflet thickness of at least 5 mm, had substantially higher rates of IE, significant mitral regurgitation, and need for valve replacement than patients with nonclassic MVP [8-10].

The 2007 AHA IE prevention guidelines shifted away from prophylaxis in moderate-risk conditions such as MVP, concluding that only a small number of IE cases could be prevented through three antibiotic administrations before dental procedures [5]. Concerns regarding antibiotic resistance, adverse drug reactions, and cost contributed to this more restrictive approach. Current recommendations limit prophylaxis to the four highest-risk groups: patients with prosthetic cardiac valves, prior IE, certain congenital heart diseases, and cardiac transplant recipients with valvular disease [5,7].

Despite these recommendations, MVP remains associated with an elevated risk of IE compared to the general population. Several studies have shown that the presence of mitral regurgitation, leaflet redundancy, and leaflet thickening are important predictors of IE risk [8-10]. MVP has also been associated with complications, including arrhythmias, heart failure progression, and structural valvular abnormalities beyond infective endocarditis [11]. 

This case raises the question of whether selected MVP patients with leaflet thickening and mitral regurgitation should undergo individualized risk stratification when considering antibiotic prophylaxis before dental procedures [12]. This case does not challenge current guideline recommendations but highlights the importance of continued evaluation of risk stratification in intermediate-risk populations. Although broad prophylaxis for all MVP patients is unlikely to be beneficial, there may be a subset of moderate-risk patients who warrant closer consideration, particularly when additional risk factors such as leaflet thickening, poor dentition, or significant mitral regurgitation are present.

## Conclusions

This case highlights the potential severity of IE in patients with MVP, even in the absence of current guideline-based indications for antibiotic prophylaxis. The severity of this presentation is likely influenced by the virulence of *Staphylococcus aureus*, which is known to cause aggressive IE. Our patient developed fulminant MSSA endocarditis after a recent tooth extraction, resulting in septic emboli, annular abscess formation, conduction abnormalities, cardiomyopathy, and urgent mitral valve replacement. Although current recommendations appropriately limit prophylaxis to the highest-risk cardiac conditions, patients with mitral valve prolapse with leaflet thickening and mitral regurgitation may represent a subgroup at increased risk for severe complications. This case highlights the need for continued investigation into risk stratification in patients with MVP and leaflet abnormalities who develop IE.
